# Synergistic Analgesia of Duloxetine and Celecoxib in the Mouse Formalin Test: A Combination Analysis

**DOI:** 10.1371/journal.pone.0076603

**Published:** 2013-10-07

**Authors:** Yong-Hai Sun, Yu-Lin Dong, Yu-Tong Wang, Guo-Li Zhao, Gui-Jun Lu, Jing Yang, Sheng-Xi Wu, Ze-Xu Gu, Wen Wang

**Affiliations:** 1 Anesthesia and Operation Center, Department of Anesthesiology, Chinese PLA General Hospital, Beijing, P. R. China; 2 Department of Anatomy, Histology and Embryology & K.K. Leung Brain Research Centre, Preclinical School of Medicine, Fourth Military Medical University, Xi’an, P. R. China; 3 Department of Emergency, Xi’jing Hospital, Fourth Military Medical University, Xi’an, P. R. China; 4 Department of Orthodontics, School of Stomatology, Fourth Military Medical University, Xi’an, P. R. China; University of Würzburg, Germany

## Abstract

Duloxetine, a serotonin and noradrenaline reuptake inhibitor, and celecoxib, a non-steroidal anti-inflammatory drug, are commonly used analgesics for persistent pain, however with moderate gastrointestinal side effects or analgesia tolerance. One promising analgesic strategy is to give a combined prescription, allowing the maximal or equal efficacy with fewer side effects. In the current study, the efficacy and side effects of combined administration of duloxetine and celecoxib were tested in the mouse formalin pain model. The subcutaneous (s.c.) injection of formalin into the left hindpaw induced significant somatic and emotional pain evaluated by the biphasic spontaneous flinching of the injected hindpaw and interphase ultrasonic vocalizations (USVs) during the 1 h after formalin injection, respectively. Pretreatment with intraperitoneal (i.p.) injection of duloxetine or celecoxib at 1 h before formalin injection induced the dose-dependent inhibition on the second but not first phase pain responses. Combined administration of duloxetine and celecoxib showed significant analgesia for the second phase pain responses. Combination analgesia on the first phase was observed only with higher dose combination. A statistical difference between the theoretical and experimental ED_50_ for the second phase pain responses was observed, which indicated synergistic interaction of the two drugs. Concerning the emotional pain responses revealed with USVs, we assumed that the antinociceptive effects were almost completely derived from duloxetine, since celecoxib was ineffective when administered alone or reduced the dosage of duloxetine when given in combination. Based on the above findings, acute concomitant administration of duloxetine and celecoxib showed synergism on the somatic pain behavior but not emotional pain behaviors.

## Introduction

Synergistic, additive or antagonistic interactions can be observed when two analgesics are given at the same time. Under the situation of synergistic interaction, the lower doses for each drug can be used to reach an equal or better analgesia with fewer overall side-effects derived from individual compounds [1]. To evaluate the preclinical analgesic effect, two animal models are commonly used, i.e. subcutaneous (s.c.) injection of formalin into the orofacial or hind paw to induce pain of face [2] or foot [3,4]. The two-phase pain responses are the shared features for both orofacial and hindpaw formalin tests and are regarded to be associated with two at least partially distinct mechanisms for nociception: the first phase is associated with direct stimulation of nociceptors, whereas the second phase reflects integration between peripheral (nociceptors) and central (spinal/supraspinal) signaling [5]. In the orofacial formalin test, face grooming behavior is used as the indicator for pain responses [6] and the combination analgesia of different medications have been investigated with this model [7-9]. However, there still remains debate whether face grooming is really a pain [6] or hypoalgesic response [10]. On the other hand, the spontaneous finching and licking of the injected hindpaw seem to be a reliable parameter for evaluating the biphasic pain responses induced by s.c. formalin injection and this model has been used in our previous study as well [4].

Antidepressants and non-steroidal anti-inflammatory drugs (NSAIDs) are two commonly used medications targeting different components of pain. Duloxetine, one of the new generation serotonin (5-HT)-norepinephrine reuptake inhibitor (SNRI) antidepressants, is used to treat depression and also alleviate allodynia in inflammatory [11-13] and neuropathic pain [14,15]. Duloxetine inhibits the reuptake of 5-HT and norepinephrine that are two important neurotransmitters released from the terminals of descending pain control pathways, thereby increasing their local concentrations [16,17] and promoting persistence of their analgesic effects. Although usually mild, the typical side effects for the SNRI class including nausea, dizziness, somnolence are generally observed in the patients with duloxetine treatment [18].

Celecoxib, one of the selective cyclooxygenase (COX)-2 inhibitors, has been extensively used in the treatment of osteoarthritis and rheumatoid arthritis [19,20]. This compound exhibits 3 featured biological activities -antipyretic, anti-inflammatory and analgesic [21] activities attributed to their inhibition of prostaglandin biosynthesis [22]. Moreover, other mechanisms such as activating the endogenous opioid/cannabinoid systems [23], inhibiting protein kinase C epsilon translocation to modulate TRPV1 function and inhibiting substance P synthesis and release [24] were recently suggested to be the possible contributors to celecoxib analgesia. However, the celecoxib analgesia also faces the gastrointestinal side effects [25] and tolerance as observed in a rat model of inflammatory pain [26].

Because both duloxetine and celecoxib are associated with increased risk of side effects, the synergistic effect at a lower dosage might be a better analgesic strategy. We hypothesized that there exists potential synergism between duloxetine and celecoxib. Since the analgesic mechanisms for duloxetine and celecoxib are different, the combinational using of each agent at lower doses may yield improved analgesia. Such a synergistic analgesia is not associated with some central nervous system (CNS) alteration reflected by locomotion and motor coordination impairments nor the consequence of anti-depression. Thus, in the current study, we observed the potential combination analgesic effect between duloxetine and celecoxib on the inflammatory pain induced by s.c. injection of formalin into one hindpaw of mice with isobolographic analysis.

## Materials and Methods

### Animals and drugs

Male C57BL/6 mice (about 10 weeks old) were housed in a temperature-controlled environment on a 12-h light/dark cycle with access to food and water ad libitum. All experimental procedures received prior approval from the Animal Use and Care Committee for Research and Education of the Fourth Military Medical University (Xi’an, China) (permit number: 10301), and the ethical guidelines to investigate experimental pain in conscious animals was followed. Formalin solution was bought from Si’chuan Xi’long Chemical Co. Ltd (Chengdu, China). Both duloxetine (Eli Lilly Company, USA) and celecoxib (Pifzer Pharmaceuticals LLC, USA) were purchased and freshly dissolved in sterile saline, filtered before use and delivered intraperitoneally (i.p.).

### Experimental design

According to our pilot experiment, the behavioral features of mice receiving s.c. saline injection were similar to those of naïve mice, thus, in the current study, the data obtained from the naïve mice were not included. Four experiments were designed to confirm our hypothesis. To reduce the bias introduced by the batch difference of animals, and to better control and compare the analgesia effect, we used separate vehicle group for each experiment.

Experiment 1 aimed to establish the dose-effect curve for duloxetine on the formalin induced somatic and emotional pain responses. After a 2-week acclimation, the animals were randomly assigned to one of the following groups: (1) mice receiving i.p. injection with saline then followed by s.c. injection with 25 µl of 5% formalin 1 h later (Veh group); (2) mice receiving i.p. injection with 3 mg/kg of duloxetine followed by s.c. injection with 25 µl of 5% formalin 1 h later (DUL 3 mg/kg group); (3) mice receiving i.p. injection with 10 mg/kg of duloxetine followed by s.c. injection with 25 µl of 5% formalin 1 h later (DUL 10 mg/kg group); (4) mice receiving i.p. injection of 30 mg/kg of duloxetine followed by s.c. injection with 25 µl of 5% formalin 1 h later (DUL 30 mg/kg group); (5) mice receiving i.p. injection with 60 mg/kg of duloxetine followed by s.c. injection with 25 µl of 5% formalin 1 h later (DUL 60 mg/kg group).

Experiment 2 aimed to establish the dose-effect curve for celecoxib on the formalin induced somatic and emotional pain responses. After a 2-week acclimation period, the animals were randomly assigned to one of the following groups: (1) mice receiving i.p. injection with saline followed by s.c. injection with 25 µl of 5% formalin 1 h later (Veh group); (2) mice receiving i.p. injection with 5 mg/kg of celecoxib followed by s.c. injection with 25 µl of 5% formalin 1 h later (CEL 5 mg/kg group); (3) mice receiving i.p. injection with 10 mg/kg of celecoxib followed by s.c. injection with 25 µl of 5% formalin 1 h later (CEL 10 mg/kg group); (4) mice receiving i.p. injection with 20 mg/kg of celecoxib followed by s.c. injection with 25 µl of 5% formalin 1 h later (CEL 20 mg/kg group); (5) mice receiving i.p. injection of 40 mg/kg of celecoxib followed by s.c. injection with 25 µl of 5% formalin 1 h later (CEL 40 mg/kg group).

Experiment 3 aimed to establish the dose-effect curve for the combination analgesia on the formalin induced somatic and emotional pain responses. After a 2-week acclimation period, the animals were randomly assigned to one of the following groups: (1) mice receiving i.p. injection with saline followed by s.c. injection with 25 µl of 5% formalin 1 h later (Veh group); (2) mice receiving i.p. injection with duloxetine and celecoxib at the effective dose ratio of 1:1 which theoretically induces 5% of pain inhibition (DUL&CEL 1 group); (3) mice receiving i.p. injection with duloxetine and celecoxib at the effective dose ratio of 1:1 which theoretically induces 10% of pain inhibition (DUL&CEL 2 group); (4) mice receiving i.p. injection of duloxetine and celecoxib at the effective dose ratio of 1:1 which theoretically induces 20% of pain inhibition (DUL&CEL 4 group); (5) mice receiving i.p. injection with duloxetine and celecoxib at the effective dose ratio of 1:1 which theoretically induces 40% of pain inhibition (DUL&CEL 8 group).

All the animals from the above-mentioned experiments were video- and audio- recorded for the later off line analysis during the 1 h time window.

Experiment 4 aimed to rule out the possibility that the analgesia is caused by the anti-depression effect of medications, and evaluate the possible CNS alterations that may contribute to the pain behaviors observations for duloxetine, celecoxib or their combination, respectively. Based on the ED_50_ values calculated from the dose-effect curves for duloxetine, celecoxib and their combination, the mice were randomly divided into the following groups: (1) mice receiving i.p. injection with saline (Veh group); (2) mice receiving i.p. injection with duloxetine at the dose of 30 mg/kg body weight (DUL 30 group); (3) mice receiving i.p. injection with celecoxib at the dose of 20 mg/kg body weight (CEL 20 group); (4) mice receiving i.p. injection with a combination dose of duloxetine and celecoxib (11.738 and 7.964 mg/kg for duloxetine and celecoxib, respectively) (DUL+CEL group). At 1 h after the i.p. injection, mice from these groups underwent rotarod, open field (OF) and elevated plus maze (EPM) tests.

### Sample size calculation

According to our pilot experiments, the area under curves (AUCs) for the second phase of vehicle and 60 mg/kg of duloxetine treated groups were about 1650 and 890, respectively, with the standard deviation of about 400. By using these data and the on line statistical power calculation tool (http://www.stat.ubc.ca/~rollin/stats/ssize/n2.html), the calculated statistical power for the second phase response from 6 mice (per group) was 0.91 (P < 0.05). When comparing the difference between vehicle and 30 mg/kg of duloxetine treated groups, such a sample size can give statistical powers of 0.78 for the second phase responses. Thus, six mice were assigned to each treatment for all the behavioral experiments.

### Formalin test

The formalin test was used to induce the somatic (flinching or licking the injected hind paw) and emotional responses (USVs emission). After the mouse’s acclimation to the testing chamber for about 20 min, twenty-five µl of the 5% formalin solution (dissolved in saline) was s.c. injected into the plantar surface of the left hind paw using a microsyringe (Hamilton Co., NV, USA) attached to a 30-G needle. After formalin administration, the mice were returned to the observing cage and the video- and audio-recordings were performed for 60 min, as described below.

All the behavioral observations were performed in a low illuminated sound-proof room. A sound-attenuated clear Perspex testing cage (25*25*40 cm) was fitted with a reverse video camera to record video for offline behavioral analysis. A trained observer conducted the behavioral analysis of the video recordings to determine the somatic pain responses induced by formalin. The observer was trained to provide a similar rating performance (at the 95% confidence limit) for each behavior during the tests of different animals. The pain behaviors were manually recorded with a stop watch by retrieving spontaneous flinching or licking of the injected hindpaw from the recorded videos. According to our previous report, spontaneous flinching and licking of the injected hindpaw have the similar validity in reflexing somatic pain [4], thus we only evaluated the spontaneous flinching of the injected hindpaw in the current study.

The recording of USVs was done using a mini-3 Bat Detector (Ultravox, Noldus Technology, Wageningen, The Netherlands), consisting of an audio filter and an ADAD converter and a personal computer with an analysis software (Ultravox 2.0, Noldus Technology). The ultrasound detector was positioned above the containing cage and set to detect frequencies of 22 kHz with an amplitude filter setting of 4 to minimize background noise [27]. The total number and duration of USVs were recorded for every 5-min period during the 60-min recordings for each mouse. Environmental noise levels were standardized to minimize their influence on ultrasound recording.

### EPM test

EPM test was done according to our previous reports [28,29] with some modifications for mice. Briefly, the Plexiglas apparatus consisted of a plus-shaped platform elevated 50 cm from the floor. Two of the opposing arms (30 cm*5 cm) were enclosed by 25 cm-high side and end walls (closed arms, CA), whereas the other two arms had no walls (open arms, OA). Mice were placed individually into the center (neutral) zone of the maze, facing an OA and were allowed to explore the maze for a 5-min period. The number of open and closed arm entries and time spent in the open and closed arms were recorded. Animals were considered to be in the open or closed arms only when all four paws crossed out of the neutral zone. The EPM relies on the animal’s natural fear of open spaces, and the percent of time spent in OA (OA time %) and percent of OA entries (OA entries %) are believed to be measurements of general anxiety level. OA time% was calculated by taking the time spent in the OA and dividing it by the sum of the time spent in the open and closed arms. OA entries% was calculated by taking the number of OA entries and dividing it by the sum of the entries into both open and closed arms (Shanghai Mobiledatum Information Technology Co., Ltd, Shanghai, China).

### OF test

Mice were placed at the center of a cubic chamber [470 mm (W) × 470 mm (H) × 470 mm (D)]. The total distance that the animal traveled in 15 min was measured by an automated analyzing system (Shanghai Mobiledatum Information Technology Co., Ltd). This distance was used as a parameter for the mice locomotion and the percent of time spent in the center area (center time %) is used to evaluate depression levels. All animals were habituated to the testing room for 20 min before the start of each session. The test room was dimly illuminated with indirect white lighting, as mice are nocturnal and their natural exploratory behavior is hindered in well-illuminated conditions.

### Rotarod test

Motor coordination and balance were determined using a standard mouse rotarod (Shanghai Mobiledatum Information Technology Co., Ltd) which provides an accelerating rotational speed from 4 rpm at the start of the test to a maximal speed of 24 rpm within 120 seconds. Only one training for each animal was performed at 1 h after i.p. administration of reagents (vehicle or drugs) by placing mice on the rotating drums (3 cm diameter) and measuring how long each animal was able to maintain its balance on the rod. The latency to fall down the rotating drums was determined automatically by a timer that is triggered off by the circuit switched on due to the fell mouse stopped LED signal. A cutoff latency of 120 s was used for all rotarod assessments.

### Dose-effect curve and ED_50_ calculation

The duloxetine, celecoxib and their combination dosages were transformed into logarithm dose and the non-line fit was performed so as to build the dose-effect curve. Based on the dose-effect cure, the ED_50_s of duloxetine and celecoxib on analgesia were calculated. The reliability of ED_50_ calculated from a specific dose-effect curve can be evaluated by the slope factor returned by the GraphPad Prism version 5.01 for Windows (San Diego California USA, www.graphpad.com).

### Isobolographic analysis

An isobolographic analysis was performed to characterize drug interaction according to the method originally described by Tallarida [1]. Both duloxetine and celecoxib did not reach a valuable percentage of antinociception in the first phase, thus they were considered as an “inactive” drug for this phase. In the second phase, both drugs achieved comparable levels of antinociception so that ED_50_ values were used to obtain a theoretical dose-response curve for a fixed-ratio combination of duloxetine and celecoxib [1].

A theoretical ED_50add_ was calculated based on the real ED_50_s for each drug under the consummation that there is only additive interaction between the two drugs. Subsequently, an experimental dose response curve was obtained by treating animals with one of the following combination doses: ED_50add/10_, ED50_add*2/10_, ED_50add*4/10_ and ED_50add*8/10_ in a fixed-ratio of 1:1 for duloxetine and celecoxib. Based on this dose-response curve, the ED_50_ of combination can be calculated and termed as ED_50comb_. An ED_50comb_ less than ED_50add_ suggest a synergistic effect of these two medications. However, the case for the emotional pain responses revealed by USVs was different from the somatic responses in that only duloxetine but not celecoxib showed efficacy. The ED_50_ of duloxetine for USVs was higher than that for the second phase somatic responses, thus, we just applied the similar combination regime used for somatic pain and could not come to the conclusion whether or not duloxetine and celecoxib has a combination analgesia for emotional pain response of the formalin model.

### Statistical analysis

The results were expressed as mean value ± standard error of the mean (SEM). In the formalin test, when comparing the somatic pain responses, data from the first phase and the second phase were considered independently; when comparing the emotional pain responses, data obtained during 1 h were pooled together. The AUC of individual animal for formalin pain response curves (somatic and USVs) and the data sets for OF, EPM and rotarod tests were group pooled and One-way ANOVA with Dunnett’s post hoc test was performed using GraphPad Prism version 5.01 for Windows (Graph Pad Software, San Diego California USA, www.graphpad.com). 

## Results

### 1: Effect of i.p. duloxetine on the formalin induced pain responses

#### Somatic pain responses revealed by the spontaneous flinching of the injected hindpaw

**Figure 1:**
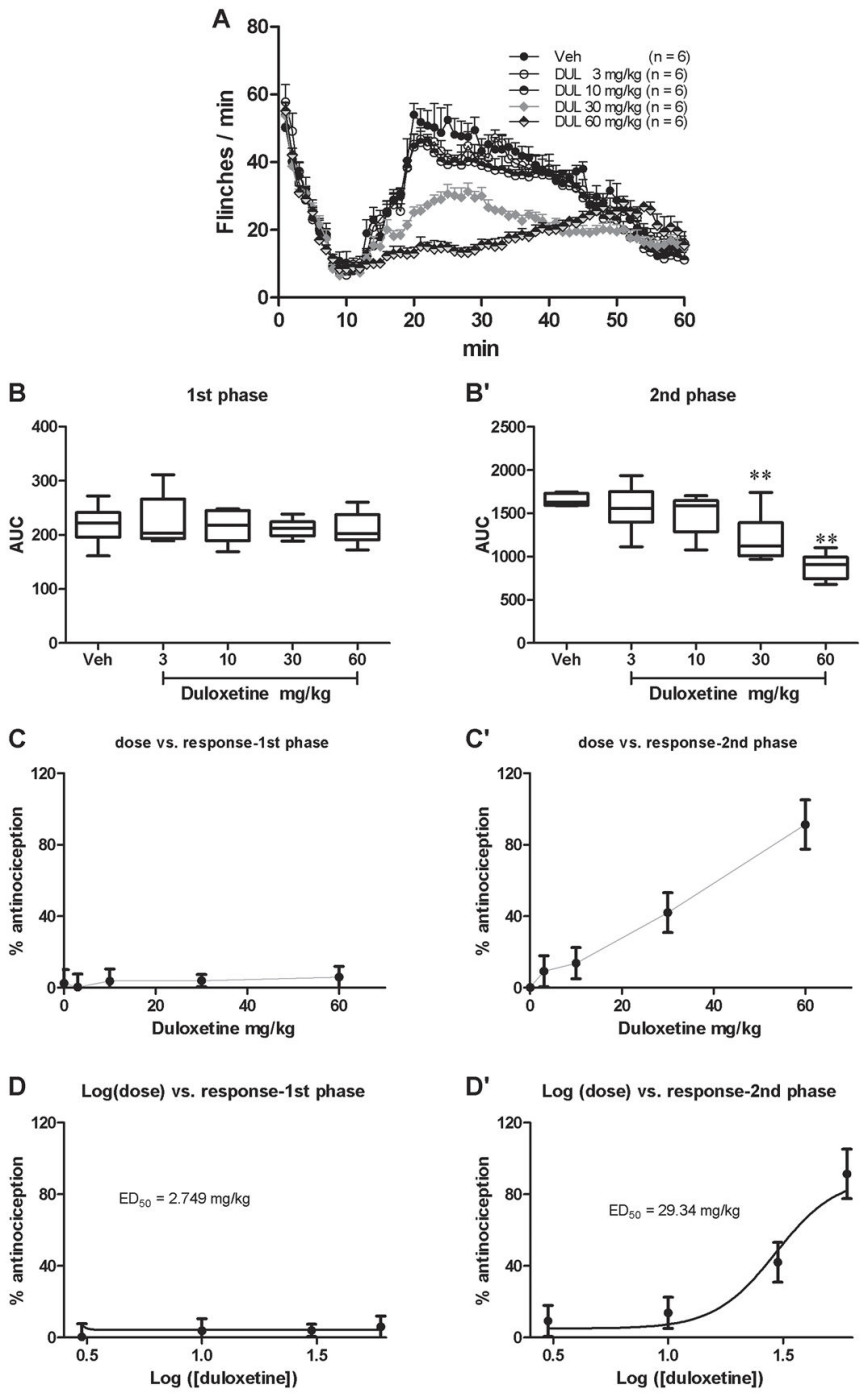
Duloxetine dose-dependently inhibited formalin induced spontaneous flinching of the injected hind paw. Spontaneous flinchings during 60 min after s.c. formalin injection from different groups were shown in A. The AUCs for different groups were calculated to perform statistical analysis on the first (B) and second (B’) phases. The dose-effect or log (dose)-effect curves for duloxetine’s analgesic effects were shown in C and D (first phase) or C’ and D’ (second phase).

An obvious biphasic flinching response can be induced by the s.c. injection of formalin (Figure **1A**). Pretreatment with i.p. duloxetine significantly affected the second but not the first phase flinchings(1). *First phase*. There was no group difference in the first phase flinchings [Figure **1A and 1B**; one way ANOVA (between-subject factor: treatment) F(4, 29) = 0.1865, p = 0.9432]. Dunnett’s *post hoc* test also revealed no group difference within the 5 groups. The effect of duloxetine on the first phase flinchings was calculated based on the log (dose) vs response curve (Figure **1D**) transformed from the dose vs response curve (Figure **1C**). However, because of the zero effect of duloxetine on the first phase pain responses, the ED_50_ value could not be retrieved(2). *Second phase*. There was a significant group difference in the second phase flinchings [Figure **1A and 1B’**; one way ANOVA (between-subject factor: treatment) F(4, 29) = 12.39, p < 0.01]. Dunnett’s *post hoc* test also revealed group difference between 30 mg/kg (P < 0.05) or 60 mg/kg (P < 0.01) with vehicle treatments. There was no significant difference between 3, or 10 mg/kg and vehicle treatment groups (P > 0.05). The effect of duloxetine on the second phase flinching was calculated based on the log (dose) *vs* response curve (Figure **1D’**) transformed from the dose vs response curve (Figure **1C’**). The ED_50_ of duloxetine on the second phase flinching was 29.34 mg/kg.

#### Emotional pain responses revealed by USVs

**Figure 2:**
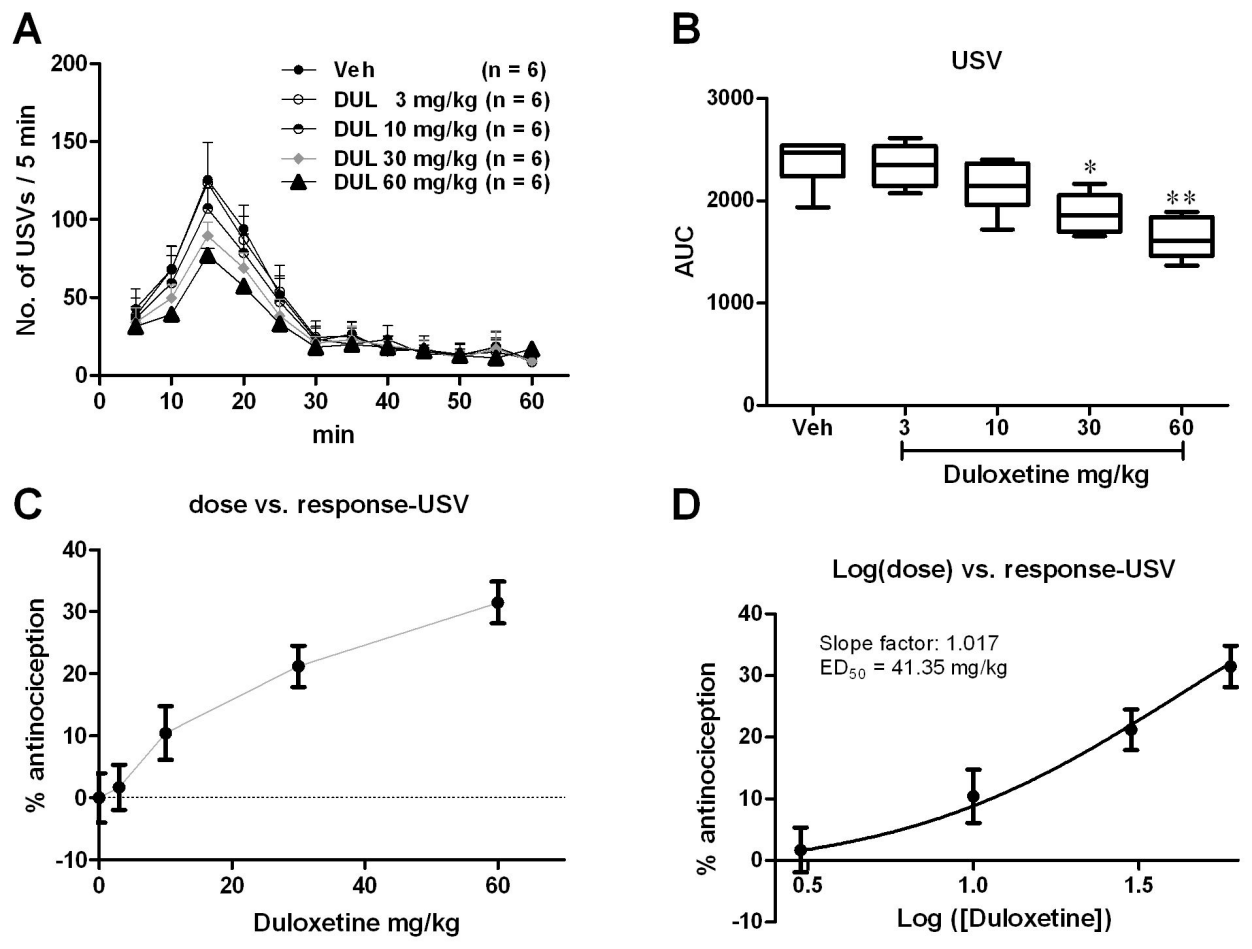
Duloxetine dose-dependently inhibited formalin induced USVs especially during the interphase (10-25 min after formalin injection). USVs curves from different groups were shown in A. The AUCs for different groups were calculated to perform statistical analysis (B). The dose-effect or log (dose)-effect curves for duloxetine’s analgesic effects were shown in C and D.

According to a previous report [30], USVs have been postulated to be an indicator of on-going pain and USVs during the formalin test can be used as a measure for the negative affective dimension of pain in rat. We also tested the validity of USVs in detecting emotional pain responses in mouse formalin pain model [4]. We filtered USVs at the 22 kHz (USVs are sound with frequency higher than 20 kHz) and performed the off-line statistical analysis. Consistent with the previous study made on rats, s.c. formalin injection into the mice hind paw triggered USVs with the peak during 10-20 min (Figure **2A**, interphase) after injection. There was a significant group difference in the USVs during 1 h after formalin injection [Figure **2A and 2B**; one way ANOVA (between-subject factor: treatment) F(4, 29) = 12.72, p < 0.01]. Dunnett’s *post hoc* test also revealed significant group difference between 30 mg/kg (P < 0.01) or 60 mg/kg (P < 0.01) with vehicle treatments. There was no significant difference between 3 or 10 mg/kg and vehicle treatment groups (P > 0.05). The effect of duloxetine on the USVs was calculated based on the log (dose) vs response curve (Figure **2D**) transformed from the dose vs response curve (Figure **2C**). The ED_50_ of duloxetine on the USVs was 41.35 mg/kg.

### 2: Effect of i.p. celecoxib on the formalin induced pain responses

#### Somatic pain responses revealed by the spontaneous flinching of the injected hindpaw

**Figure 3:**
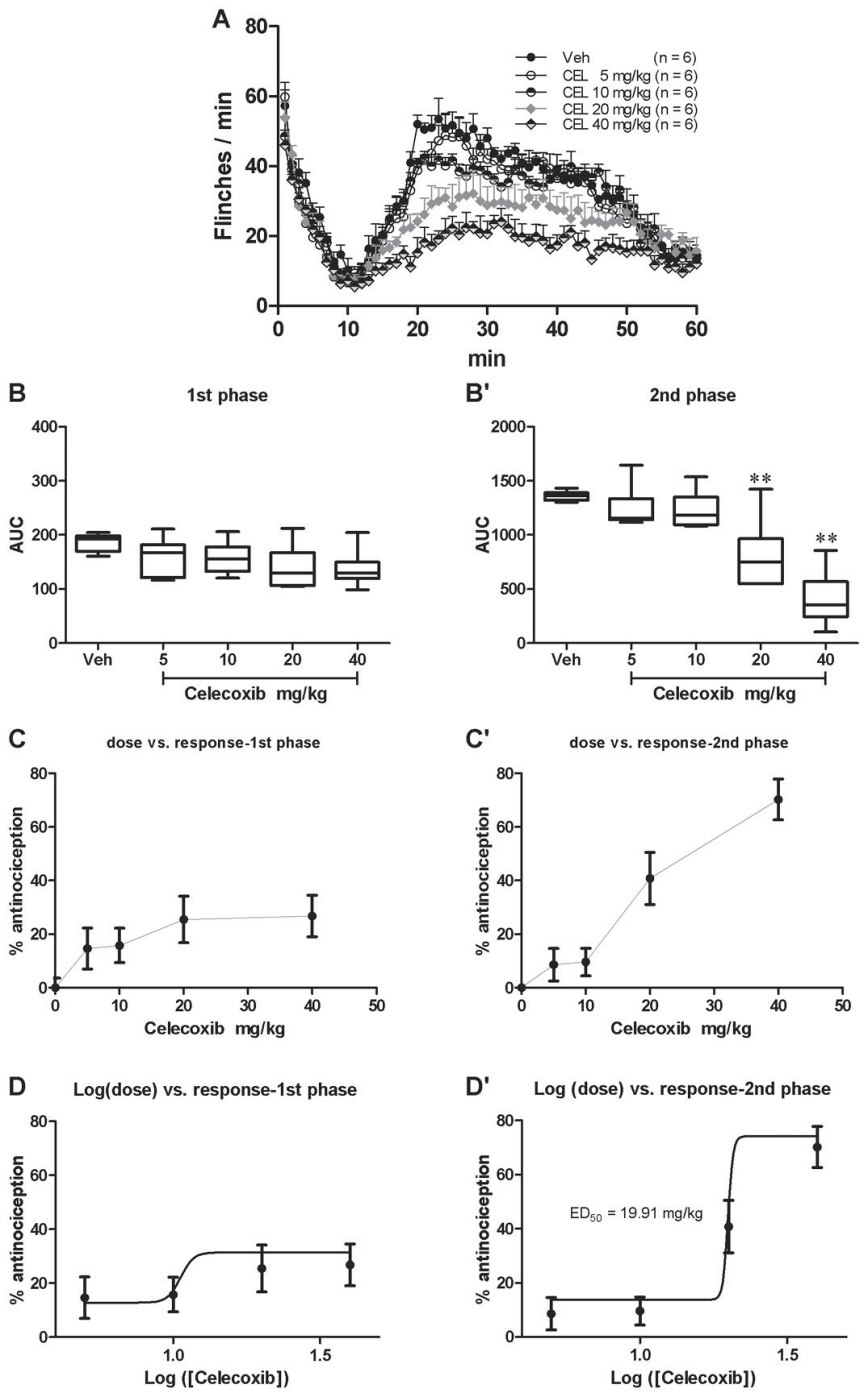
Celecoxib dose-dependently inhibited formalin induced spontaneous flinching of the injected hind paw. Spontaneous flinchings during 60 min after s.c. formalin injection from different groups were shown in A. The AUCs for different groups were calculated to perform statistical analysis on the first (B) and second (B’) phases. The dose-effect or log (dose)-effect curves for celecoxib’s analgesic effects were shown in C and D (first phase) or C’ and D’ (second phase).

Pretreatment with i.p. celecoxib significantly affected the second but not the first phase flinchings(1). *First phase*. There was no group difference in the first phase flinchings [Figure 3A and 3B; one way ANOVA (between-subject factor: treatment) F(4, 29) = 2.321, p = 0.0846]. Dunnett’s *post hoc* test also revealed no group difference within the 5 groups. The effect of celecoxib on the first phase flinchings was calculated based on the log (dose) *vs* response curve (Figure **3D**) from the dose vs response curve (Figure **3C**). However, because of the zero effect of celecoxib on the first phase pain responses, the ED_50_ value could not be retrieved(2). *Second phase*. There was a significant group difference in the second phase flinchings [Figure **3A and 3B’**; one way ANOVA (between-subject factor: treatment) F(4, 29) = 19.62, p < 0.01]. Dunnett’s *post hoc* test also revealed group difference between 20 mg/kg (P < 0.05) or 40 mg/kg (P < 0.01) with vehicle treatments. There was no significant difference between 5 or 10 mg/kg and vehicle treatment groups (P > 0.05). The effect of celecoxib on the second phase flinchings was calculated based on the log (dose) vs response curve (Figure **3D’**) from the dose *vs* response curve (Figure **3C’**). The ED_50_ of celecoxib on the second phase flinchings was 19.91 mg/kg.

#### Emotional pain responses revealed by USVs

**Figure 4:**
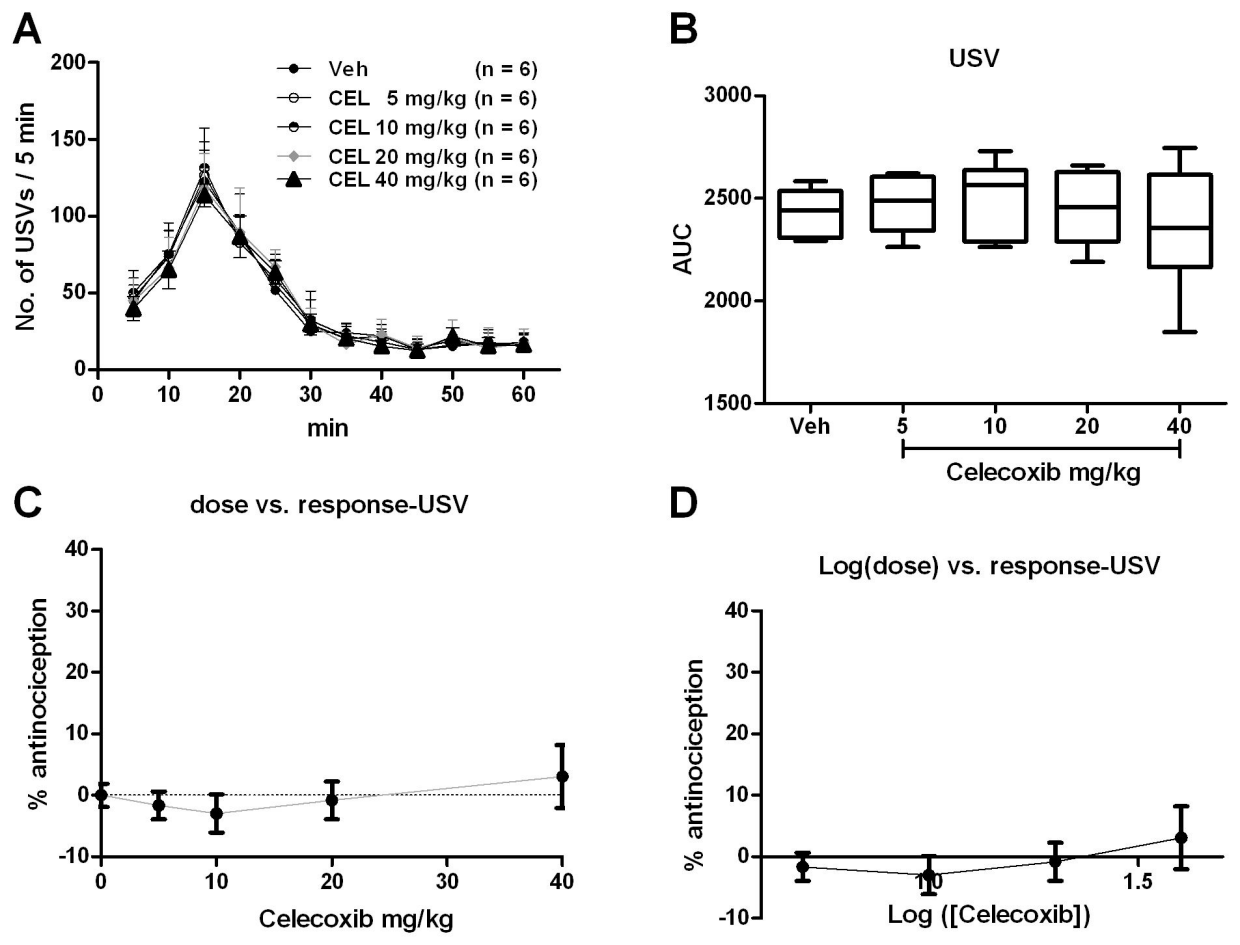
Celecoxib had no effects on the formalin induced USVs. USVs curves from different groups were shown in A. The AUCs for different groups were calculated to perform statistical analysis (B). The dose-effect or log (dose)-effect curves for celecoxib’s analgesic effects were shown in C and D.

There was no significant group difference in the USVs during 1 h after formalin injection [Figure **4A and 4B**; one way ANOVA (between-subject factor: treatment) F(4, 29) = 0.4721, p = 0.7557]. Dunnett’s *post hoc* test also revealed no significant group difference between 5, 10, 20 or 40 mg/kg with vehicle treatments (P > 0.05). The effect of celecoxib on the USVs was calculated based on the log (dose) *vs* response curve (Figure **4D**) from the dose *vs* response curve (Figure **4C**). However, because of the zero effect of celecoxib on the formalin induced USVs, the ED_50_ value could not be calculated.

### 3: Effect of duloxetine and celecoxib combination on the formalin induced pain responses

#### Interaction analysis for the somatic pain responses

**Figure 5:**
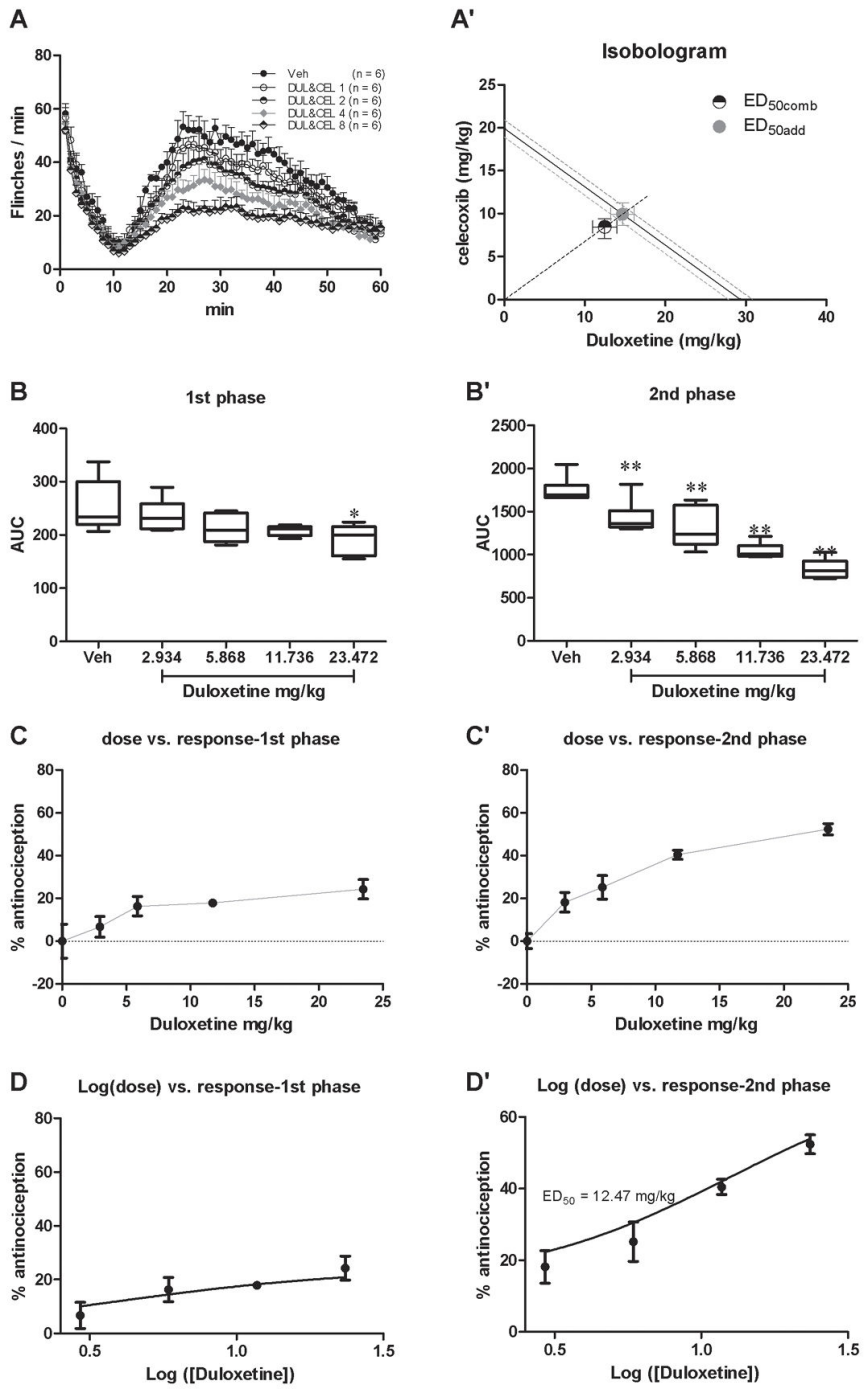
The duloxetine and celecoxib combinations dose-dependently inhibited formalin induced spontaneous flinching of the injected hindpaw. Spontaneous flinchings during 60 min after s.c. formalin injection from different groups were shown in A. The calculated ED_50_ (ED_50add_) and actual ED50 for combination analgesia (ED_50comb_) were shown in A’. The AUCs for different groups were calculated to perform statistical analysis on the first (B) and second (B’) phases. The dose-effect or log (dose)-effect curves for combination analgesic effects were shown in C and D (first phase) or C’ and D’ (second phase).

Due to the different action profiles for duloxetine and celecoxib, interaction parameters were calculated on the basis of the antinociceptive effects exerted by the two drugs for the second phase. Middle dose response curves of both compounds were linear, thus, a composite additive curve was constructed (Figure **5A’**). Additive regression allowed us to calculate theoretical ED_50_ for a fixed-ratio (1:1) combination of duloxetine and celecoxib (ED_50add_= 14.67 duloxetine + 9.955 celecoxib). The dose regime designed to investigate the experimental ED_50comb_ included the following combinations: 2.934 duloxetine + 1.991 celecoxib (DUL&CEL1), 5.868 duloxetine + 3.982 celecoxib (DUL&CEL2), 11.736 duloxetine + 7.964 celecoxib (DUL&CEL4) and 23.472 duloxetine + 15.928 celecoxib (DUL&CEL8).

Experimental data on the effect of combination doses are showed in Figure 5. There was significant group difference in the first phase flinchings during 1 h after formalin injection [Figure **5A and 5B**; one way ANOVA (between-subject factor: treatment) F(4, 29) = 3.595, p = 0.0190]. Dunnett’s *post hoc* test revealed that this difference derived from comparison between DUL&CEL8 and vehicle treatments (Figure **5B, 5C** **and** 5D, P < 0.05). Considering the zero analgesic effect for both medications even under high dosages on the first phase pain responses, this suggested that there might be combination analgesia for both drugs on the first phase pain responses, however, we did not test this concept with isobolographic analysis. Meanwhile, There was significant group difference in the second phase flinchings during 1 h after formalin injection [Figure **5A and 5B’**; one way ANOVA (between-subject factor: treatment) F(4, 29) = 27.41, p <0.01]. Dunnett’s *post hoc* test revealed that these difference derived from the significant difference between DUL&CEL1 (P < 0.05), 2 (P < 0.01), 4 (P < 0.01) or 8 (P < 0.01) groups and vehicle treatment group. The experimental ED_50comb_ calculated from these dose-response curves (Figures **5C’** and **5D’**) for the second phase pain responses was 12.47 duloxetine + 8.46 celecoxib. Isobolographic analysis of duloxetine and celecoxib combination effect on the second phase pain responses showed the ED_50comb_ was smaller than the lower (95%) range of ED_50add_, suggesting that the interaction between the two drugs was synergistic (Figure **5A’**).

#### Interaction analysis for the emotional pain responses

**Figure 6:**
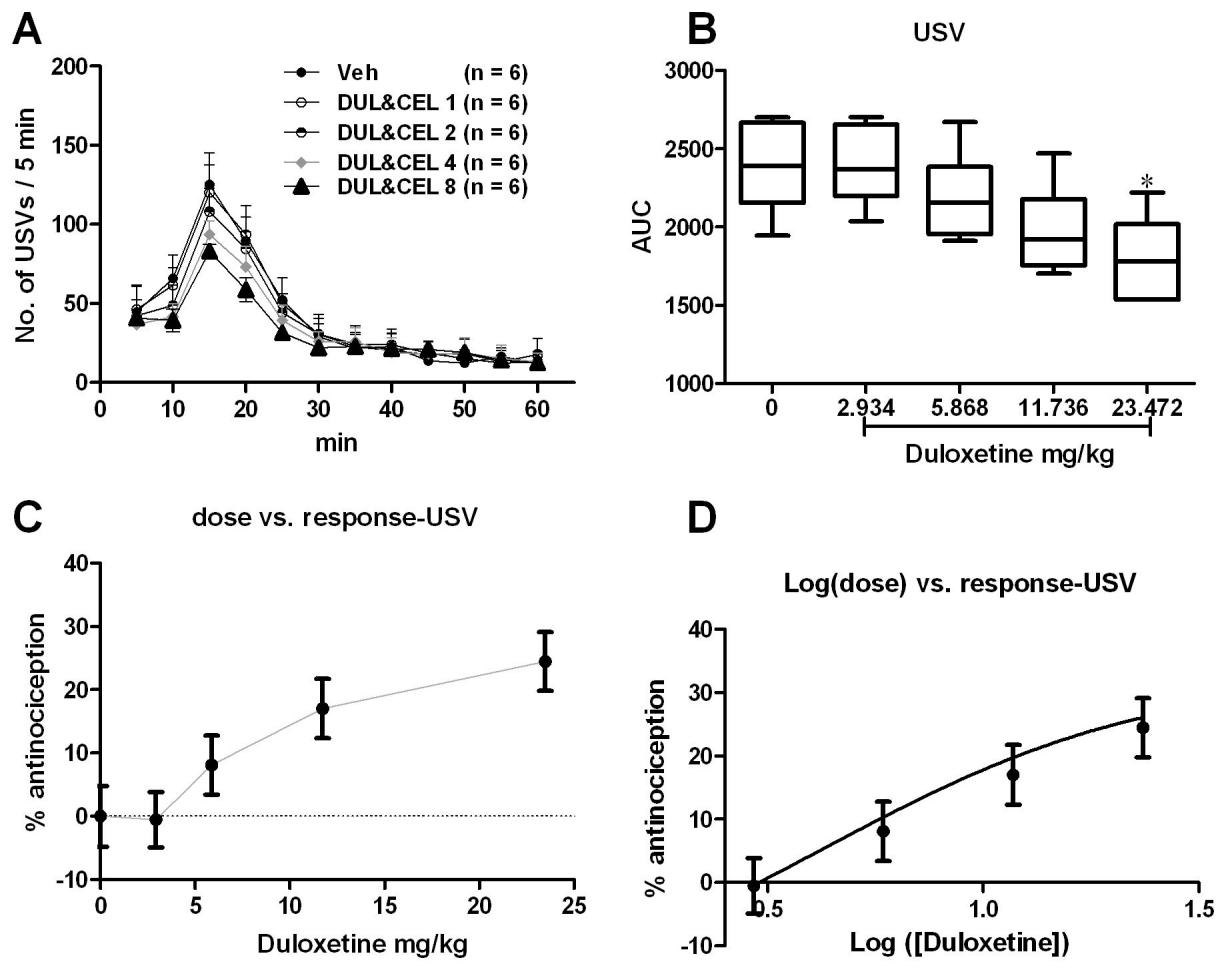
The combination had significant analgesia effect only under high dose on the formalin induced USVs. USVs curves from different groups were shown in A. The AUCs for different groups were calculated to perform statistical analysis (B). The dose-effect or log (dose)-effect curves for the combination analgesic effects were shown in C and D.

Because celecoxib didn’t show significant analgesic effect on the emotional pain responses indicated by the USVs, we could not get an ED_50_ for celecoxib on the emotional pain responses nor perform the followed isobolographic analysis. Thus, the predetermined dose regime of DUL&CEL 1, 2, 4 and 8 were used to detect the potential combination analgesia on the emotional pain responses (Figure **6**).

There was a significant group difference in the USVs during 1 h after formalin injection [Figure **6A and 6B**; one way ANOVA (between-subject factor: treatment) F(4, 29) = 5.476, p =0.0026]. Dunnett’s *post hoc* test revealed that these differences derived from the comparison between DUL&CEL8 and vehicle treatments (P < 0.05). The effect of combinational medication on the USVs was calculated based on the log (dose) *vs* response curve (Figure **6D**) transformed from the dose *vs* response curve (Figure **6C**). The analgesia effect reached the ceiling of about 25% antinociception at a dosage of DUL&CEL8 which is far below the dosage of 41.35 mg/kg for duloxetine that induced about 50% antinociception. Thus, we could not retrieve the ED_50comb_ in this experimental setting. However, we have the strong impression that there was no synergistic effect when these two drugs were used in combination because about half of the ED_50_ dosage for duloxetine in the combination (23.472 mg/kg) induced about 25% antinociception which was expected by using 20.675 mg/kg duloxetine alone. This fact means that the analgesia on USVs at the combination of DUL&CEL 8 was derived from duloxetine itself but not the synergy or addition from celecoxib. However, this conclusion still remains open and future isobolographic analysis with the theoretical and experimental ED_50_ of combination based on the ED_50_ for USVs is needed.

### 4: Acute effect of i.p. duloxetine, celecoxib or their combination on some CNS functions

**Figure 7:**
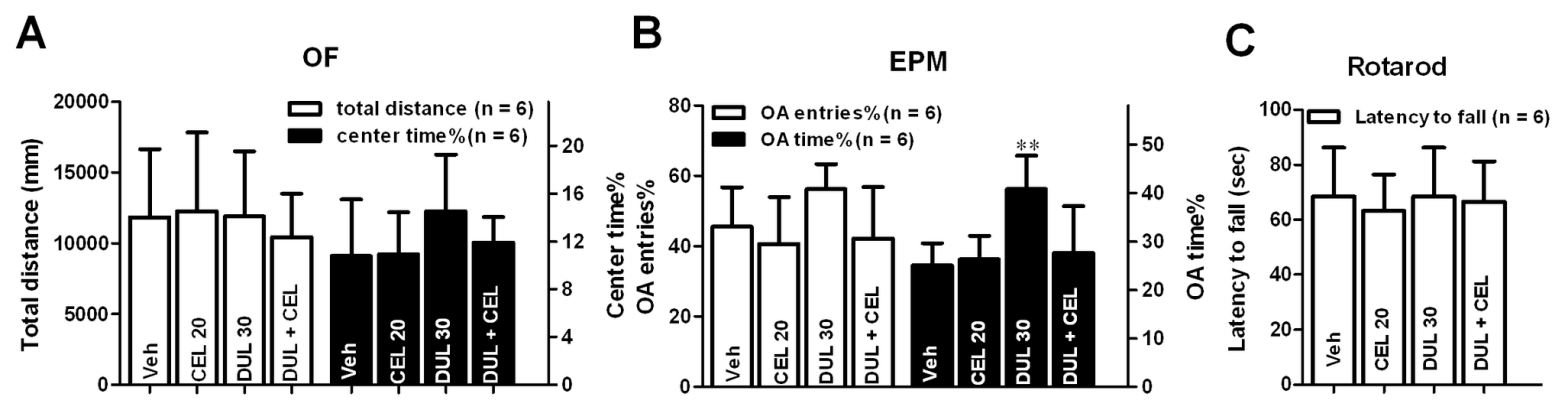
Duloxetine (DUL 30), celecoxib (CEL 20) or their combination (DUL + CEL) did not affect animals’ locomotion, anxiety/depression level and motor coordination. Total distance traveled and center time% in OF (A), OA entries% and OA time% in EPM (B) and latency to fall in rotarod test (C) were used to detect the locomotion, anxiety/depression and motor coordination, respectively.

The pain behaviors are often accompanied with increased anxiety and reduced locomotion [31]. On the other hand, emotional change such as anxiety/depression can affect the formalin induced inflammatory pain responses [32,33]. The analgesic measurement of two tested drugs and their combinations in the current study might be affected by the alteration of mice anxiety/depressive status or locomotion. Thus we tested the possible CNS functional alterations at 1 h after i.p. drug treatments to naïve mice on the locomotion, anxiety and motor coordination by using OF, EPM and rotarod tests, respectively. For this purpose, the dosages for individual drug or drug combination close to the individual ED_50_ were selected, ie. 30 mg/kg for duloxetine, 20 mg/kg for celecoxib and DUL + CEL (11.738 and 7.964 mg/kg for duloxetine and celecoxib, respectively) for the combination. There was no significant group difference in the locomotion revealed by the total distance traveled during the 15 min recording time in OF [Figure **7A**; one way ANOVA (between-subject factor: treatment) F(3, 23) = 0.189, p = 0.902]. Dunnett’s *post hoc* test also revealed no significant group difference between CEL 20, DUL 30 or DUL + CEL and vehicle treatments. In the OF test, there was no significant group difference in the percentage of center time [Figure **7A**; one way ANOVA (between-subject factor: treatment) F(3, 23) = 1.180, p = 0.342], which indicated no difference in their depression-like behaviors. Insignificant difference in the anxiety-like behaviors was also indicated by the OA entries% [Figure **7B**; one way ANOVA (between-subject factor: treatment) F(3, 23) = 2.113, p = 0.131]. However there was significant difference for the OA time% [Figure **7B**; one way ANOVA (between-subject factor: treatment) F(3, 23) = 06.989, p < 0.01]. Dunnett’s *post hoc* test revealed that this significant difference was derived from the DUL 30 group (P < 0.01). Furthermore, i.p. drug pretreatment did not alter the motor coordination [Figure **7C**; one way ANOVA (between-subject factor: treatment) F(3, 23) = 0.148, p = 0.930].

## Discussion

To our knowledge, this is the first report showing combination analgesia for duloxetine and celecoxib on inflammatory pain induced by s.c. injection of formalin into the hind paw. Although duloxetine and celecoxib have been commonly used in the treatment of pain, especially for inflammatory or neuropathic pain, the gastrointestinal side effects and the desensitization phenomenon of celecoxib as well as the SNRIs side effects limit the clinic application of both drugs. The combination analgesia observed in the current study may help open the venue of a new analgesic strategy that induces significant analgesia with reduced side effects.

Our results further suggested that the combination analgesia of duloxetine and celecoxib is not caused by the alterations of higher brain functions, which is at least true in the current acute inflammatory pain model because the combination dosage did not alter the higher brain functions significantly.

### 1: Duloxetine and celecoxib combination may be another new analgesic strategy

Duloxetine has long been used in the treatment of diabetic neuropathic pain [34,35] besides its antidepressant application [36,37]. It is also believed that its analgesic effects originate from attenuating the emotional pain responses so as to reduce the patients subjective pain score and improve the life quality [38,39]. However, duloxetine has to be administered for some period to reach a certain serum concentration before becoming effective for depression or neuropathic pain. In the current study, we offered evidence that 1 hour administration is already long enough for duloxetine to induce analgesic effects on both somatic and emotional pain responses in the acute inflammatory pain model. This finding is somehow inconsistent with previous ones and the underlying mechanisms for the acute analgesia of duloxetine need to be investigated in the future. Celecoxib shows analgesia effect when administered acutely [40] or chronically [41]. Our data suggested that acutely administered celecoxib induced analgesia on the somatic pain responses but not emotional pain responses and that the single administration of celecoxib did not change the stress related behaviors evaluated with OF and EPM. This is partially different with a previous report focusing on the effect of celecoxib on tooth movement related stress and pain responses [42]. The possible explanation may be that different animal models were used. The dental model may be considered more of a “pure” inflammatory model, whereas injection of formalin may be more a “mixed” picture.

In the current study, the combination analgesia for duloxetine and celecoxib was observed for the second phase somatic but not the emotional pain responses in the formalin model. Although this combination is not ideal for attenuating both somatic and emotional pain responses, it has obvious advantages over the individual medications. First, smaller dosages of each agent may be used to reach equal or better efficacy. Second, the side effects decreased significantly. Third, because the effective dosage for each drug is lower than the normal level, the potential drug interactions with other simultaneously used drugs are reduced. In this sense, the combination of duloxetine and celecoxib may serve as a new analgesic strategy.

### 2: Duloxetine, celecoxib or their combination favors the second more than the first phase formalin pain responses via the spinal mechanisms

Our study suggests that duloxetine, celecoxib or their combination pretreatment mainly attenuate the second phase formalin pain responses more than that of the first phase and this effect may be preferentially mediated by spinal versus supra-spinal mechanisms. According to conventional views, the first phase formalin response is predominantly due to peripheral sensitization that involves the sensitization of peripheral nociceptors via direct activation of transient receptor potential ankyrin (TRPA)-1 receptors [43]; while the second phase of the formalin response is associated with stimulation of TRPA1 [44] and also with the development of an inflammatory response triggered by many mediators such as interleukin (IL)-1β, IL-6, IL-8, tumor-necrosis factor (TNF)-α [45], PGE and NO [46]. Celecoxib is an inhibitor of PGE generation and thus inhibits nociceptive responses.

Meanwhile, the second phase formalin response is believed to be the consequence of central sensitization that largely involves spinal cord neurons [43,47,48] or primary sensory neurons [49]. These spinal cord neurons and primary sensory neurons are under the regulation of the descending pain control system originating from supra-spinal structures including the rostral ventral medulla that is a primary source of serotonergic inputs to the spinal dorsal horn [50] and the dorsolateral pontine tegmentum that is a source of noradrenergic input to the spinal cord [51,52]. Serotonin and norepinephrine released from the descending control system to the spinal cord bind with relevant post- or presynaptic receptors and inhibit the neurotransmission between primary afferent fibers and projection neurons [53-56]. In this way, pain responses were attenuated; duloxetine may particularly modulate intrathecal 5-HT_2A_ receptor in neuropathic pain [57].

Theoretically, i.p. injected duloxetine and celecoxib can reach the higher brain structures and spinal cord to enroll serotonergic, noradrenergic and prostanoid systems to render analgesia for the second phase pain responses. However, the current acute administration may not induce a sustained increase of drug concentration in the higher brain structures, but the involved primary nociceptive integrating system might be mainly involved in the analgesia and the relatively rapid increase of 5-HT and NE levels at the spinal level is a feasible mechanism underlying the analgesia effect. The fact that the used dosage and treatment schedule for either duloxetine or the combination of duloxetine and celecoxib induced minor effects on the emotional pain or the higher brain functions also suggests that the higher brain structures may not play a major role in the analgesia after this acute treatment.

Furthermore, our findings have some clinical significance. The lack of anti-nociceptive effects of either drug in the first phase of the formalin test but the potent effects in the second phase suggests that these drugs modulate not only nociceptive but also hyperalgesic mechanisms, which are relevant to pathological insults such as surgery. Indeed, multimodal analgesia (including preemptive treatment) is an important topic in managing post-surgical pain and effective targeting of hyperalgesic mechanisms is generally considered to be the best way to prevent chronic pain [58,59].

In summary, our present study offers experimental evidence that i.p. treatment with duloxetine and celecoxib in a fixed ratio combination yields a synergistic analgesia after formalin injection and can attenuate pain but not affect the emotional status or locomotion significantly. The combination of duloxetine and celecoxib may serve as a therapeutic option for pain control with minimal severe side effects. However, the efficacy and side effects for chronic or repeated combination treatment to provide sustained analgesia needs to be investigated in the future.

### 3: Gap between animal research and human application

The scientific community that relies heavily on animal studies is the major sources for clinicians to decide the human dose for a certain medication in the human clinical trials. However, it is a usual case that a drug that works well in animals is ostensibly not effective in humans. One often-ignored explanation for drug ineffectiveness is the inappropriate translation of a drug dose from one animal species to another. The calculations for determining starting dose in humans as extrapolated from animals should use the more appropriate normalization of body surface area (BSA) than the body weight alone [60].

By using the following formula [1], we calculated the human equivalent dose (HED) based on our animal study.

HED (mg/kg) = (mouse dose (mg/kg) * mouse K_*m*_)/human K_*m*_ (1)

K_*m*_ factor for mouse and adult human being are 3 and 37, respectively.

The translated ED_50_ of duloxetine or celecoxib for an adult people with 60 kg body weight is 142.74 or 96.86 for a day, respectively. However, the maximal dosage of duloxetine in the treatment of diabetic neuropathic pain is 120 mg/day which is still lower than our current HED for inflammatory pain. On the other hand, the effective dosage of celecoxib for osteoarthritis pain in adult human being is 200 mg/day that is higher than our current HED for inflammatory pain. The current dosage regimes for both duloxetine and celecoxib are also commonly used in different neuropharmacological studies [61-63], thus, one possible explanation for the discrepancy between animal studies and human trials might be that mouse and human being demonstrated different sensitivity for serotonin-noradrenaline and COX2 systems. However, this explanation needs further experimental evidence.

Drs. Sun Y-H, Dong Y-L and Wang Y-T contributed equally to this manuscript as first authors. Drs. Gu Z-X and Wang W co-corresponded this project. This manuscript received editing service from YouthMed Science and Technology limited company.
